# Commentary: A quantitative analysis of artificial intelligence research in cervical cancer: a bibliometric approach utilizing CiteSpace and VOSviewer

**DOI:** 10.3389/fonc.2025.1666369

**Published:** 2025-10-15

**Authors:** Zai'xiang Zhang, Xianlin Luo, Hua Zhao

**Affiliations:** Hubei University of Chinese Medicine Affiliated Gong’an Hospital of Traditional Chinese Medicine, Jingzhou, China

**Keywords:** cervical cancer, artificial intelligence, bibliometrics, CiteSpace, quantitative analysis

## 1 Introduction

In recent years, the exponential growth in biomedical literature has garnered significant attention for bibliometrics as a method capable of quantitatively and qualitatively analyzing research trends and hotspots within a given discipline. We read with great interest the publication by Zhao et al. (1), titled “A quantitative analysis of artificial intelligence research in cervical cancer: a bibliometric approach utilizing CiteSpace and VOSviewer,” which has been published in the issue of Frontiers in Oncology. We highly support and appreciate the researchers’ work and thank them for their contributions in the field.

## 2 Commentary and discussion

Using bibliometrics, this study conducted an in-depth analysis of the existing publications on the application of artificial intelligence (AI) in the field of cervical cancer. The analysis reveals that AI technology is playing an increasingly important role in several key aspects of cervical cancer, including in early diagnosis screening, treatment plan formulation, prognostic evaluation, and image analysis. However, we identified several points that require clarification and correction.

**Figure 1:**
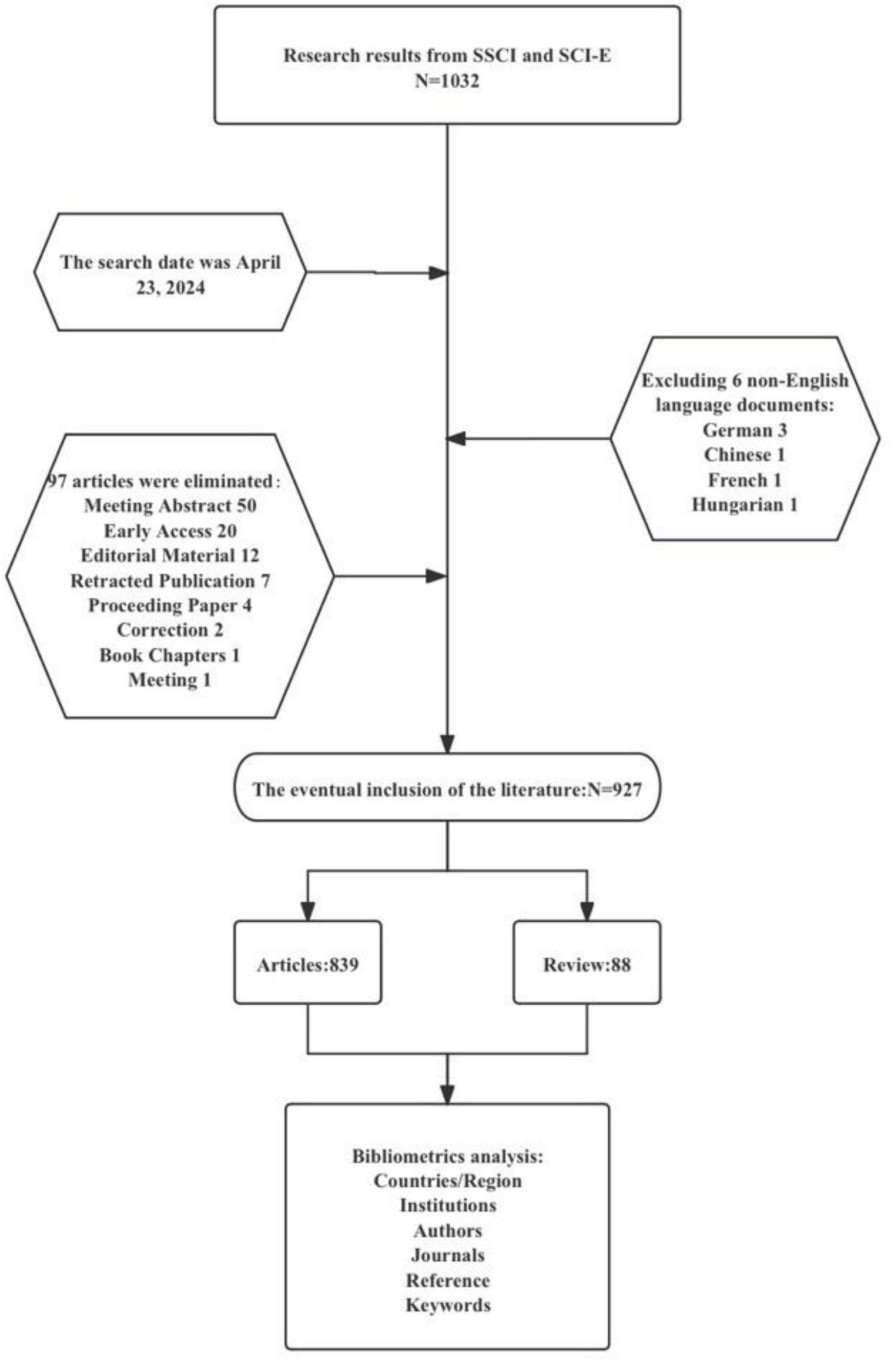
A flow chart of the retrieval process in this study.

Firstly, inconsistencies in the literature search and screening numbers: The manuscript states in multiple sections that 927 publications were ultimately included. However, **Figure 1** (a flowchart of the retrieval process) indicates that 97 publications were excluded for not being original research or review articles and six were excluded for being non-English publications. This results in a total of 103 excluded publications. Therefore, the initial number of records identified must logically be 927 (included) + 103 (excluded) = 1,030 records. This calculation conflicts with data in Table 1, which state, “research results from SSCI and SCI-E (*N* = 1,032),” and the “Manual screening process” section, which states, “we preliminarily screened 1,027 relevant papers.” We recommend that the authors verify and correct these inconsistencies (1,032 in Table 1 and 1,027 in the text) to align with the flowchart data, which imply an initial count of 1,030 records.

Secondly, omission in the institution ranking (Table 2): The “Productive institutions analysis” section states, “According to the data in Table 2, the top three institutions in terms of TLS are the National Cancer Institute of the National Institutes of Health (TLS = 141), Southern Medical University (TLS = 81), and the Chinese Academy of Medical Sciences (TLS = 80).” However, examination of Table 2 revealed that Peking Union Medical College also has a TLS = 80, placing it equally third with the Chinese Academy of Medical Sciences. We recommend that the authors amend this sentence to acknowledge Peking Union Medical College as co-third place.

**Figure 2:**
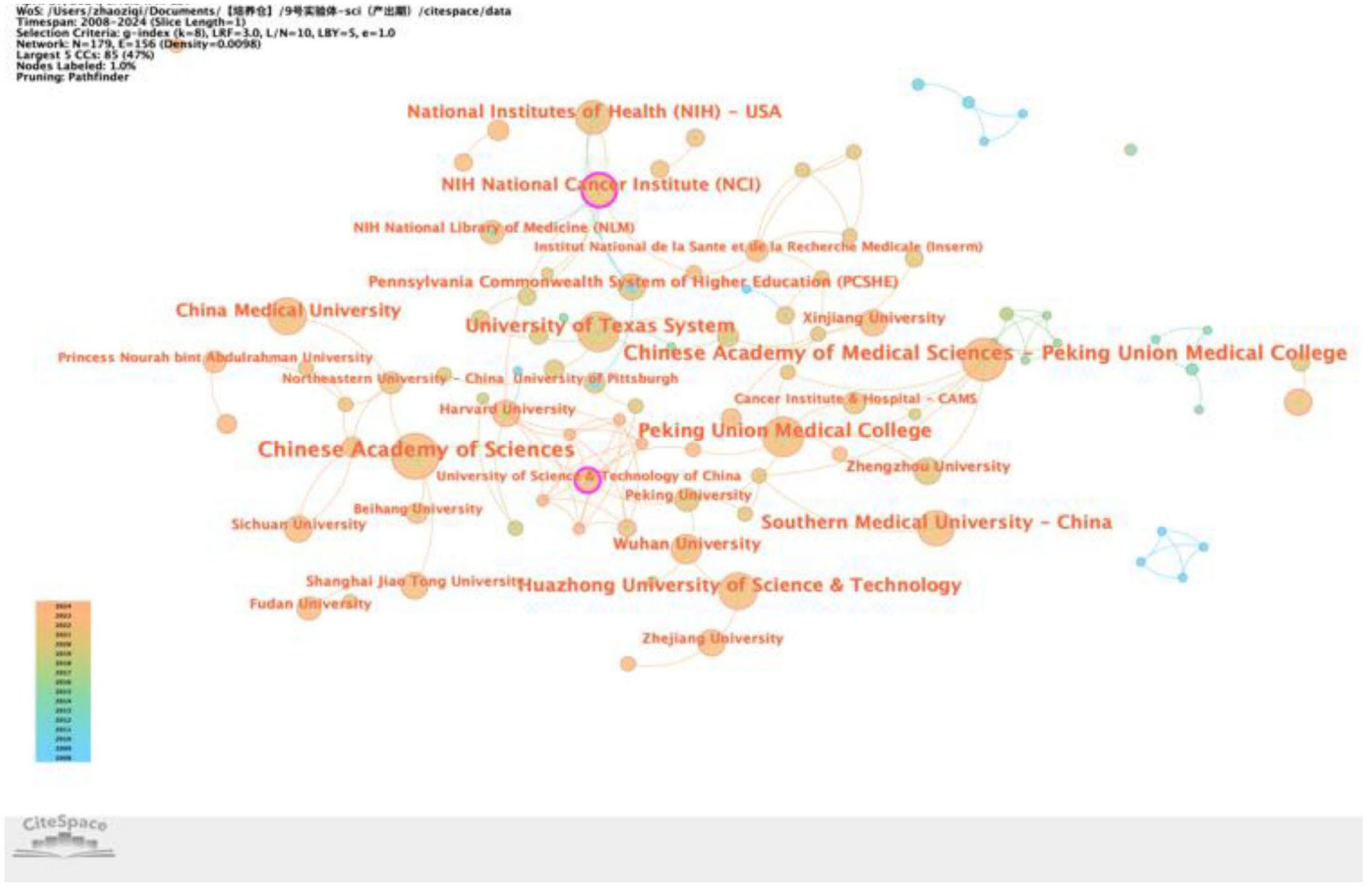
Centrality map of institutional collaboration.

**Figure 3:**
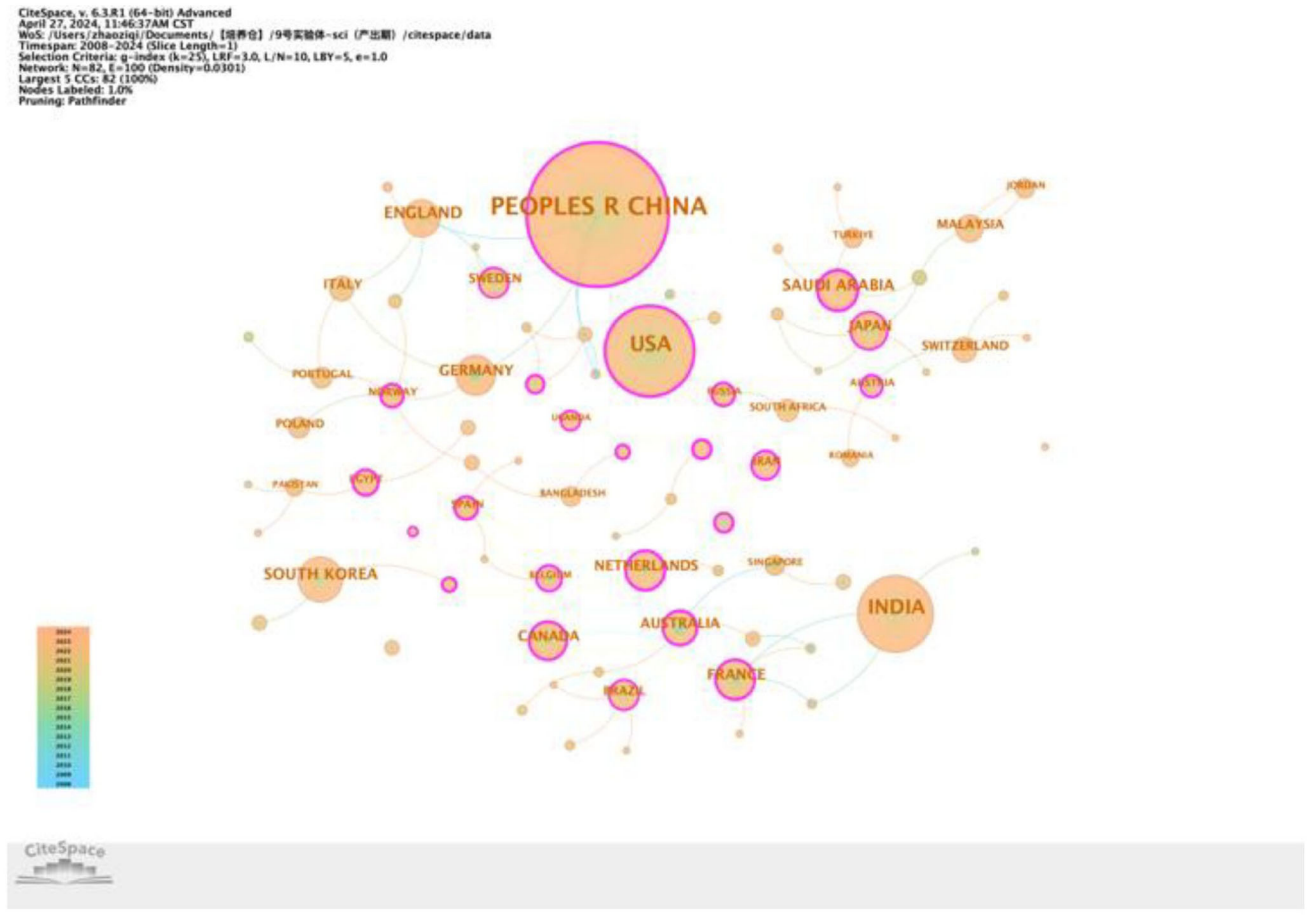
Map of the centrality of national cooperation.

Thirdly, concerns regarding the CiteSpace g-index parameter (*k*-value): The methodology for generating the institutional collaboration network (**Figure 2**) states, “…setting a time span from 2008 to 2024, with a 1-year slicing length, using institutions as the node type, and setting the g-index to *k* = 8.” We noted that **Figure 3** uses a g-index of *k* = 25, while all other figures generated with CiteSpace in this study have been reported to use *k* = 10. It is understood that, in CiteSpace, the g-index parameter (*k*) controls the number of nodes selected within each time slice. A higher *k*-value (*k* = 25) retains more nodes, while a lower *k*-value (*k* = 8 or *k* = 10) retains fewer nodes. We are concerned that the use of *k* = 8 (**Figure 2**) and *k* = 10 (majority of the other figures) may be significantly lower than the common default or standard value (frequently *k* = 25), potentially excluding too many nodes. This raises the question: Could these relatively low *k*-values have resulted in an incomplete representation of the networks, failing to fully capture and interpret the relevant collaborative structures or knowledge domains? We recommend that the authors justify their choice of these specific *k*-values and discuss whether this parameter selection might have impacted the comprehensiveness of their network visualizations and analyses.
